# Effect of Different Surface Modifications of Acrylic Teeth and Thermocycling on Shear Bond Strength to Polycarbonate Denture Base Material

**DOI:** 10.1155/2022/9855836

**Published:** 2022-02-08

**Authors:** Humam M. Al-Somaiday, Aula Kamal Rafeeq, Manar E. Al-Samaray

**Affiliations:** Department of Prosthodontics, College of Dentistry, Mustansiriyah University, Baghdad, Iraq

## Abstract

During prosthodontic clinical practice, the most commonly reported type of failure is the debonding of teeth to the denture base. Incompatible surface conditions at the tooth/denture base interface result in a lack of bonding. This study aimed to study the influence of different surface modifications of acrylic teeth and thermocycling on shear bond strength to polycarbonate denture base material. Eighty cylinder-shaped samples were fabricated. The tested samples were divided into 4 groups (*n* = 20). Group A represents the control group, group B represents the mechanical modification of the tooth, while group C and group D represent the chemical treatment of the tooth with ethyl acetate and bonding agent, respectively. Each group was further subdivided into 2 categories depending on the thermocycling procedure (*N* = 10). All samples were tested for shear bond strength tests. A computer-controlled universal testing machine performed the shear bond test at a 0.5 mm/min crosshead speed. Three-way ANOVA (*P*=0.05) was used for the statistical analysis of the data. Results show that shear bond strength was significantly affected by the surface treatment, whether it is mechanical or chemical (*P* ≤ 0.01) (B > D > C) compared with a control group (A). However, thermocycling has a nonsignificant decrease in the bond strength values in all experimental groups (*P* > 0.05) (B > D > C > A). The mechanical treatment by creating retentive holes (B) provides better results than the chemical surface treatment with a bonding agent and ethyl acetate (D and C, respectively). This study concluded that various surface conditioning methods affect the bonding strength of acrylic teeth and polycarbonate denture base material with no effect of thermocycling.

## 1. Introduction

Even though dental implants have garnered a lot of attention and have had a lot of success in treating patients who have lost all or some teeth, dentures are still the best option for treating patients with partial or complete loss of their dentition [1].

Polymeric materials are used in the fabrication of complete and partial dentures. Synthetic resin is a wide class of polymers. Phenolic resins, acrylic resins, epoxy resins, unsaturated polyester resins, vinyl ester resins, thermoplastic resins, thermosetting resins, and elastomers are among the prevalent resins [2].

Acrylic resin, polymethyl methacrylate (PMMA), is a widely used denture base material owing to its favourable properties such as pleasing aesthetics, adaptation, and stability over denture foundation area, accessible laboratory and clinical manipulation, and inexpensive equipment [3]. Artificial acrylic teeth have long been used to fabricate complete dentures and partial dentures and, more recently, in implant dentistry. They are favoured over porcelain teeth due to their characteristics, including ease of adjustment, little financial cost, improved bonding denture resin, and higher shock absorbability [4]. In their study, Huggett et al. [5] revealed that about one-third of all denture repairs carried out by three dental laboratories were due to detachment of acrylic teeth from the denture base [6].

Tooth detachment from the acrylic denture base may result from factors such as the orientation of functional forces and the available area for bonding with the denture base (ridge lap area). Wax or tin foil substitute contaminant of the denture teeth is considered the principal cause of adhesive failure [7].

Recently, considerable development in the science of biomaterials has been revealed, and new materials have been developed. Selecting appropriate denture base and acrylic teeth is necessary to minimise teeth debonding and prosthesis fractures [8]. The use of thermoplastic resins in dentistry, such as acetyl resins, polycarbonate resins, and nylons (polyamides), has increased [9].

Poly(bisphenol A) carbonate is an amorphous polymer. It is transparent and light in weight, with appropriate mechanical properties. It has good dimensional stability, excellent impact resistance, and high plastic deformation without a crack. Due to its high thermal resistance, it can maintain its properties through a wide range of temperature changes (from 140°C to −20°C). [10].

Numerous studies have reported the shear bond strength between acrylic teeth and acrylic denture bases. Multiple retention systems for denture teeth have been mentioned in the literature, such as macromechanical (pins or diatoric undercuts), micromechanical (high-energy abrasion), or chemical adhesion methods [11–14].

Primarily, ethyl acetate is used as a solvent and diluent due to its low cost, toxicity, and agreeable odour. On the contrary, the bonding agent (Thermo fusing liquid) consists mainly of acetone and isopropyl alcohol and is also considered a solvent used to improve the adhesion between denture surfaces and the tooth surface [15].

Some studies [4, 8, 16] have mentioned an adverse effect of thermocycling on shear bond strength between acrylic teeth and denture resins.

Various studies have discussed the shear bonding strength between acrylic teeth and acrylic denture base resin, considering surface treatment modifications, thermocycling, polymerisation method, teeth type, etc. Still, none reported this property between acrylic teeth and the polycarbonate denture base. This study was conducted to investigate the effect of thermocycling and various surface modifications on shear bond strength of acrylic teeth to polycarbonate denture base material after considering the following proposed research hypotheses where

1. The null hypothesis (H0) proposes that neither surface modifications nor thermocycling affects the shear bond strength between the acrylic teeth and the polycarbonate denture base material
2. The alternative hypothesis (H1) assumed that either surface modifications or thermocycling affects the shear bond strength between the acrylic teeth and the polycarbonate denture base material

## 2. Materials and Methods

Some of the materials used in this study are listed in Table 1.

### 2.1. Sample Grouping

This study investigated the shear bond strength between the modified acrylic teeth and the polycarbonate denture base material before and after thermocycling. Eighty cylinder-shaped samples were fabricated. Testing samples were divided into 4 groups (*n* = 20). Group A represents the control group, group B represents the mechanical modification of the tooth, while group C and group D represent the chemical treatment of the tooth with ethyl acetate and thermo fusing liquid (bonding agent), respectively. Each group was further subdivided into 2 categories depending on the thermocycling procedure (Table 2).

### 2.2. Surface Modifications of Acrylic Teeth

Right maxillary central incisors were used in this study to prepare the samples. Different surface treatments were made to the teeth. The resultant groups were as follows:

  Group A (control): no treatment
  Group B: mechanical preparation of 2 mm-width and 0.5 mm-depth retentive holes on the ridge lap portion of the tooth
  Group C: chemical treatment using ethyl acetate
  Group D: chemical treatment by applying a bonding agent

Both chemically treated groups received the chemical agents after the wax elimination stage.

### 2.3. Acrylic Patterns and Copper Tube Preparation

**Figure 1:**
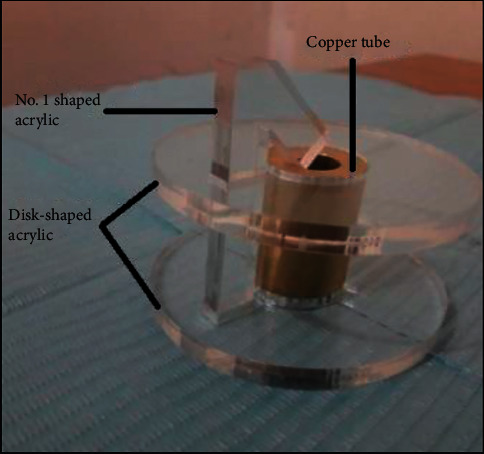
Acrylic patterns (disk-shaped and no. 1 shaped transparent acrylic) and copper tube assembly.

To fabricate the test samples with accurate positioning of the acrylic teeth, three parts were assembled. The first and the second parts were disk-shaped and no. 1 shaped transparent acrylic (acrylic patterns), while the third was the copper tube. These parts were fabricated as follows:

(1) The laser cutting machine used to prepare acrylic patterns (disk-shaped and no. 1 shaped transparent acrylic) was programmed using AutoCAD 2019 computer software. The following is the procedure for cutting acrylic patterns (glass-look acrylic sheets, Clairvauxles-Lacs, France):
    (i) A central hole (24.5 mm diameter) was constructed in custom-made disk-shaped transparent acrylic (71 mm diameter and 6 mm thickness) to insert the custom-cut copper tubing. A 45° angled end customised (1) shaped bar (6 mm width, 11 mm length, and 6 mm thickness) slit was produced on one side to obtain an angled end custom-made (1) shaped bar [9] (Figure 1).
    (ii) A custom-made bar is of 9.75 mm width, 62.25 mm length, and 5.75 mm thickness, approximating no. 1 shape with 45° angled end. [17] was utilised for accurate positioning of acrylic teeth (Figure 1).
(2) A customised copper tube, designed by using the turning machine, with 35 mm length, 24.5 mm diameter outer dimension, and inner hole (14 mm diameter), was used to prepare the cylindrical wax pattern (Figure 1) [9].

### 2.4. Samples' Preparation

**Figure 2:**
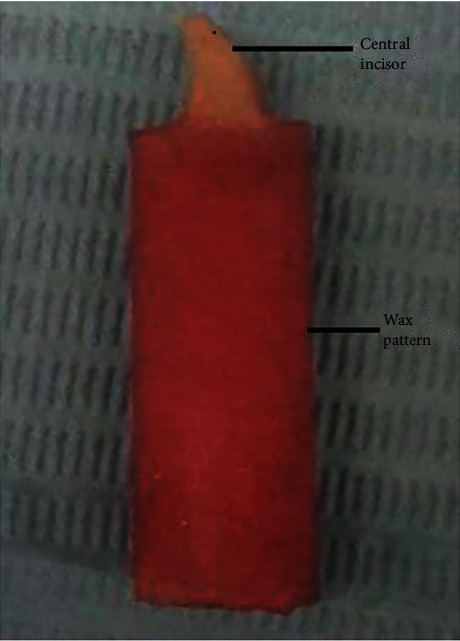
Tested sample shape.

A cylinder-shaped sample, 35 mm length and 12 mm radius, was fabricated with the acrylic tooth positioned in the centre with 45° angle 17 Testing samples were prepared as follows:

1. Coat the acrylic patterns and copper tube with a separating medium and leave them to dry.
2. Assemble the copper tube and the acrylic disk-shaped part.
3. Fill the copper tube hole with molten wax with the aid of a funnel. A wax plug was used to close the lower end of the tube to prevent the escapement of the resin during pouring.
4. For group B, mechanical preparation of 2 mm-width and 0.5 mm-depth retentive holes on the ridge lap portion of the tooth was made before incorporating the tooth into the poured wax [18]. According to manufacturer's instructions, group D teeth were roughened before applying the bonding agent.
5. Place the acrylic tooth (modified or not) into the poured wax inside the copper tube hole, and ensure the tooth's neck is plunged into the wax.
6. Assemble the no. 1 shaped bar with the preassembled parts by inserting it into the slot at the side of the disk-shaped acrylic part that holds the copper tube. With the aid of its angled end, contacting the buccal surface of the tooth, the tooth will be positioned accurately. This step will ensure the standardisation of all test samples.
7. Finally, remove the cooled wax pattern from the copper tube (Figure 2).

### 2.5. Preparation of the Mould Parts

Generally, the conventional procedure for the complete denture processing technique was followed. In the beginning, the testing samples were coated with a separating medium and allowed to dry. Then, a dental stone mixture, according to the manufacturer's instructions, was poured into the drag (metal flask lower part). Later on, invest one-half of the wax patterns into the dental stone, and wait until the dental stone's complete setting. After the dental stone was wholly set, a separating medium was used to coat the stone surface and allowed to dry.

Wax channels were connected to the wax pattern, allowing the denture base injection. The cap (the flask upper part) was adequately positioned and poured mixed dental stone under vibration. After completing the dental stone, the wax elimination process was performed by immersing the flask into a hot bath (100°C) for 10 min [19].

### 2.6. Injecting, Curing, and Finishing of the Test Samples

After the wax elimination stage, the two parts of the flask were separated, and the wax particles were removed from the mould. Both upper and lower parts of the flask were coated with a separating medium to prepare for packing.

As mentioned earlier, after wax elimination and before injecting the polycarbonate denture base material, a chemical treatment to groups C and D was made. In group C, the teeth were swabbed with ethyl acetate for 120 seconds 20 while in group D, the teeth were brushed with the bonding agent for 30 seconds, as stated in the manufacturer instruction sheet.

Polycarbonate cartridges were placed in the Deflex Mad device (Deflex, Argentina) and injected under 5–7 bar pressure and 305°C ± 10°C temperature for 15 min.

After deflasking of the test specimens, all specimens were finished and polished. Finishing was performed by using sandpaper with 120 grain size. Any excess material over the neck of the tooth should be removed with care. The next step was to immerse the samples in a rubber bowl filled with water to avoid overheating.

Testing samples were removed from water and transferred to the polishing machine to start the polishing procedure (1500 rpm) under continuous water cooling that provides a gloss surface and prevents overheating. Digital Vernier was then used to check the dimension of the sample, and those with nonstandardized measurements were discarded [9].

### 2.7. Testing Procedure

According to ADA 1999 Specification no.12 19 each testing sample should be immersed in a container filled with distilled water and stored inside the incubator (Fisher Scientific, USA) for 48 hours at 37°C before the testing procedure.

Half of the samples of each group were thermocycled using a thermocycling machine (MSCT-3, Elquip, Brazil). A total of 5,000 cycles simulating five years of oral environment were performed. After immersing each sample in 5° and 55°C (30 seconds in each) water baths, the test was performed [7].

For both groups, a computer-controlled testing machine (WDW-20, Laryee Technology Co., Ltd., Beijing, China) was used to conduct the test. A metal stud with a wedge-shaped end loaded the samples with a 50 kg load directed towards the incisal one-third of the tooth to simulate the clinical forces on the maxillary central incisor. The test was performed at a 0.6 mm/min crosshead speed and a 1000 N cell range load until tooth fracture/detachment occurred [9, 17].

To analyse the differences between the study groups, a one-way analysis of variance (ANOVA) was used. Furthermore, the least significant difference (LSD) post hoc test was used to compare the mean value of each experimental group. Statistically high significance (HS) was considered when a probability *P* value ≤0.01. *P* value ≤0.05 was deemed to be significant (S), while *P* value ≥0.05 was considered nonsignificant (NS).

IBM SPSS^®^ software (the Statistical Package for Social Sciences) (version 23.0) analysed the computerised data.

## 3. Results

Before thermocycling, statistical analysis of the test groups (B, C, and D) revealed a highly significant difference (*P* ≤ 0.01) in the shear strength value. The chemical surface treatment with ethyl acetate and the bonding agent (C and D) (266.4 N and 279.5 N), respectively, improved the bonding strength of the polycarbonate denture base with acrylic teeth when compared with the control group (A) (162.5 N). Yet, the mechanical treatment by creating retentive holes is still the best scenario (315.9 N). However, thermocycling does not significantly affect the bonding strength between acrylic teeth and polycarbonate denture base (Table 3).

## 4. Discussion

This study was performed to be an attempt to increase the clinical performance of the complete and partial dentures made from nonmetal clasp dentures, reducing the most common type of failure of these prostheses (teeth detachment). This was performed by selecting polycarbonate as denture base material and evaluating the shear bond strength of acrylic teeth to this material after various surface treatments and thermocycling.

The findings of this study indicate that the mechanical treatment had higher bond strength values when compared to other tested groups with no significant effect of thermocycling, rejecting the null hypothesis.

For the control group (A), the bonding strength between polycarbonate resin and acrylic teeth arises from partially blending melted polycarbonate with the contacted acrylic teeth. This bonding may result when the temperature increases than the PMMA glass transition. However, this is not strong enough, but it can hold the tooth in position. Besides, the high surface roughness of polycarbonate may help form a physical bond between it and the acrylic teeth [21].

However, for group B, in addition to what is explained for group A, modifying the ridge lap area with prefabricated retentive holes increases the shear bond strength by increasing the surface area and producing an undercut area. This area will prevent the separation of the material from the undercuts. This is supported by understanding the chemical structure of polycarbonate, which forms a three-dimensional cross-linked network. This structure gives polycarbonate superior flexibility and elasticity at room temperature; this is why the polycarbonate resin was found at the fracture point for group B at the neck of the tooth [22].

Before packing the resin, coating the tooth with a chemical agent has been proposed to improve the bond strength between artificial teeth and denture base [23, 24]. The bonding agents' composition should have a minimal solvent effect on the ridge lap tooth structure while allowing appropriate polymer cross-linking at the tooth-resin interface [25].

In this study, applying a chemical agent causes an increase in the bonding strength between the polycarbonate resin and acrylic teeth compared to the control group. This finding can be explained by the chemical surface treatment that causes superficial fracture growth and the creation of many pits with a diameter of 2 *μ*m. This morphologic alteration on the surface may help with mechanical retention [26]. However, this increase is more minor than reported in group B. These findings were supported by visual inspection of the broken sample pieces, which showed no remnants of the denture base material adhered to the ridge laps of the acrylic teeth.

Two chemicals were used to etch the tooth. Group C with ethyl acetate treatment shows higher results than group D with thermo fusing liquid. These findings may be attributed to the differences in the chemical composition between these products, the time of application, and the treatment procedure.

To simulate intraoral conditions, thermocycling was employed in this study. Generally, applying heat and cold alternatively may force the tooth and the denture base material to expand and contract repeatedly. As a result, this will stress the bonding area and decrease the bond strength. However, in this study, thermocycling has a nonsignificant reduction in the shear bond strength among all tested groups. Polycarbonate contracts little after injection. In addition, it does shrink more than acrylic resins, resulting in considerable dimensional stability after thermocycling [27, 28].

Furthermore, this nonsignificant difference could be attributed to the number of cycles employed. Further studies with various materials, ridge lap modifications, and several thermal processes are recommended.

## 5. Conclusions

After considering the limitations of this study, the mechanical preparation at the ridge lap area results in the highest increase in the shear bonding strength between the acrylic teeth and the polycarbonate denture base material among the tested groups. Besides, thermocycling demonstrates a nonsignificantly decreased shear bond strength between the acrylic teeth and the polycarbonate denture base material among the tested groups.

## Figures and Tables

**Table 1:** Materials used to prepare the test samples.

Material	Manufacturer
Acrylic teeth	Betastar teeth, Beta Dent, Tehran, Iran
Baseplate wax	Shanghai New Century Dental Materials Co., China
A bonding agent (Thermo fusing liquid)	Vertex-Dental, Netherlands
Ethyl acetate	Labort Fine Chem Pvt. Ltd, India
Gypsum separating solution	Isodent, SpofaDental, Czechoslovakia, Europe
Polycarbonate (extra rigid polymer M10_XR_)	Deflex, Argentina
Dental stone	Kimberlit extra hard high-density die stone, Spain

**Table 2:** Grouping of the tested samples.

Groups	Group A	Group B	Group C	Group D
Before thermocycling	10	10	10	10
After thermocycling	10	10	10	10

**Table 3:** Descriptive statistics, one-way ANOVA, and LSD values of the shear bond strength test before and after thermocycling.

Surface treatment	Before thermocycling		ANOVA *F*-test	*P* value	After thermocycling		Compared groups		*P* values
	Mean	SD			Mean	SD			
No treatment (A)	162.5	4.403	43302.158	≤0.01^*∗∗∗*^ HS	159.5	3.240	A2	A1	0.061^*∗*^
Mechanical (B)	315.9	3.725			314.5	3.240	B2	B1	0.377^*∗*^
Chemical									
Ethyl acetate (C)	266.4	3.502			264	4.197	C2	C1	0.132^*∗*^
A bonding agent (D)	279.5	2.953			276.5	4.197	D2	D1	0.063^*∗*^

^*∗*^No significant difference (*P* > 0.05). ^*∗∗*^Significant difference (*P* ≤ 0.05). ^*∗∗∗*^Highly significant difference (*P* ≤ 0.01).

## Data Availability

The data used to support the findings of this study are available from the corresponding author upon request.
